# Exploring the effect of utilising organic acid solutions in ultrasound-assisted extraction of pectin from apple pomace, and its potential for biomedical purposes

**DOI:** 10.1016/j.heliyon.2023.e17736

**Published:** 2023-06-28

**Authors:** Joel Girón-Hernández, Michelle Pazmino, Yeison Fernando Barrios-Rodríguez, Chiara Tonda Turo, Corinne Wills, Fabio Cucinotta, Maria Benlloch-Tinoco, Piergiorgio Gentile

**Affiliations:** a Department of Applied Sciences, Faculty of Health and Life Sciences, Northumbria University, NE1 8ST Newcastle upon Tyne, UK; b Department of Food Technology, Universitat Politècnica de València, Camino de Vera s/n, 46021 Valencia, Spain; c Centro Surcolombiano de Investigación en Café (CESURCAFÉ), Universidad Surcolombiana, 410007 Neiva, Colombia; d Department of Mechanical and Aerospace Engineering, Politecnico di Torino, 10129 Turin, Italy; e School of Natural and Environmental Sciences, Newcastle University, NE1 7RU Newcastle upon Tyne, UK; f School of Engineering, Newcastle University, NE1 7RU Newcastle upon Tyne, UK

**Keywords:** Apple pomace, Pectin, Ultrasound-assisted extraction, Principal component analysis, Biomaterials

## Abstract

Biomass resulting from food production represents valuable material to recover different biomolecules. In our study, we used apple pomace to obtain pectin, which is traditionally extracted using mineral acids. Our hypothesis consisted of carrying out extractions with organic acids, assisted by ultrasound, by varying processing parameters including time, temperature, and type of acid. The analytical determinations of galacturonic acid content, methoxylation and esterification degree, ζ-potential and extraction yield were used as pectin quality indicators. Pectins extracted using treatment conditions with better performance were assessed biologically *in vitro* for their potential to be used in biomedical applications. Overall, the extracted pectin presented a galacturonic acid content, methoxylation and esterification degree ranged from 19.7 to 67%, 26.8–41.4% and 58–65.2% respectively, and were negatively charged (−24.1 to −13.2 mV). It was found that factors of time and temperature greatly influenced the response variables excepting the esterification degree, while the acid type influenced the ζ-potential, methoxylation and esterification degrees. Additionally, it was seen that the longer extraction time (50 min) and higher temperature (50 **°**C) exhibited the better extraction yield (∼10.9%). Finally, the selected pectin showed high cytocompatibility up to 500 μg/mL of concentration when seeded with Neonatal Normal Human Dermal Fibroblasts.

## 1 Introduction

Valorisation of agro-industrial biowaste is a smart strategy that must be achieved through efficient and reproducible approaches, valuing green chemistry principles. Particularly, extraction and purification of bioactive compounds can impact socio-environmental demands or economic challenges [1]. Indeed, although apple crop in 2022 was affected by weather conditions in Asia, around 79 million tonnes of this fruit was produced worldwide [2]; in this scenario, value-added apple products such as juice, cider, jam and dried, account for 25–30% of the above volume, leading to a pomace biowaste mass that can reach up to 25% of the fresh fruit weight [3]. Particularly, apple pomace is a valuable material for extracting high attractive biomolecules like carbohydrates, polyphenols and triterpenes [4]. Pectin (PEC) is an interesting molecule present in vegetable cell walls and could be recovered from apple pomace and other vegetable biomass sources [5]; it is a carbohydrate polymer with plenty of applications in food sector. Traditionally, PEC has been used as gelling or thickening agent, where this stabiliser property is complemented by the attractive utilisation as a fat replacer and health-promoting functional ingredient [6]. Alternative emerging applications include the use of pectin in the biomedical and pharmaceutical industries, due to its simple and cytocompatible gelling mechanism, that can be exploited in different applications including drug and gene delivery, wound healing and tissue engineering [7]. Indeed, natural biopolymers are at the centre of materials development for biomedical and biotechnological applications based on their low-toxicity, biodegradability and biofunctional key features [8].

Current literature reports several works focused on PEC extraction from apple pomace; on a commercial scale, diverse conditions are carried out for its purpose. However, PEC is generally extracted trough water-mineral acidic solution (sulfuric, nitric, phosphoric, hydrochloric) at a pH around 1.5, where the biomass is heated at temperatures >80 °C, followed by an ethanol precipitation at different concentrations, from 70% to absolute [9,10]. Above-mentioned parameters can lead easily to equipment corrosion and environmental pollution derived from the acidic wastewater disposal [11]. Therefore, experimental studies with apple pomace or peel, have been conducted for exploring alternatives procedures to make PEC extraction process more sustainable and to enhance its recovery. In this sense, methodologies such as: organic acid extraction, application of eutectic solvents, sequential extraction, enzymatic extraction, assisting extraction with microwaves, radio frequency, ultrasounds or the combination of this methodologies have been proposed. Indeed, Cho et al. [12] have compared different acidic extractions, by using mineral and organic acids; they found that similar amounts of pectin were extracted (∼6.6%) with 1 M organic acids (tartaric, malic, citric) with an esterification degree ranged from 54 to 64.8% compared with conventional extraction (∼6.4%) by using HCl. Furthermore, a two-step slight acidic process using H_2_SO_4_ (pH 2.4) under hot stirring (100 °C) was conducted for 110 min, leading to a PEC extraction yield of ∼15%, the debris remained from the process, were used to extract cellulose-rich substances and monosaccharides, obtaining a recovery rate of 38–49% respectively [13]; this experiment represents a complete valorisation example of apple pomace; however, PEC extraction was carried out using conventional methods. Other alternative involving eutectic pre-treatments can be considered, where glycerol and lactic acid have been mixed either with choline chloride (pH 1–6.5), potassium carbonate (pH 12–14), urea or oxalic acid, leading to a final yield of extracted PEC in the range of 6–8.5% with a methoxylation degree ranged from 54 to 79%, and with an overall recovery of neutral sugars between 76 and 87% [11,14]. Nevertheless, this sequential extraction lasted more than 48 h and PEC extraction yield was not significantly high compared with findings of other authors that explored different methodologies; for example, mediating the extraction process with enzymes, it was obtained ∼7% of extraction yield, and the result did not present a much better performance when assisted with ultrasound (∼8%). Although, in the same experiment when changing the conditions to citric acid as extractant solution at pH 2.2 and microwave assisted at pH 1.8, PEC recovery was improved up to ∼23% for both conditions [10]. Recently, Zheng et al. [15] combined the use of citric acid solutions at pH (1.5–2.5) with microwave (MWAE) and radio frequency (RFAE) assisted extractions, reaching temperatures between 80 and 90 °C for 20 min. Both MWAE and RFAE procedures helped to get an extraction yield of ∼11%, that resulted in a higher performance compared with citric acid extraction at pH 2.2 as control (∼7.5% PEC recovery). Furthermore, following RFAE method, higher content of galacturonic acid content (∼63%) and esterification degree (∼66%) were reported compared with MWAE and citric acid control (∼41 and ∼51% for the galacturonic acid, and ∼54 and ∼59% for the esterification degree respectively). Thus, microwaved and radiofrequency techniques can substantially reduce duration of the extraction; however, their execution could result demanding because batch processing is required [16]; additionally, microwaves generate uneven heating due to high temperature, that might cause degradation of the components in the outermost areas of the mass volume being extracted [17].

Finally, Dranca et al. [18] proposed the use of citric acid solutions, assisted with ultrasound, up to 30 min of extraction process. They found out that at maximum ultrasound amplitude and lower pH, PEC extraction yield and degree of esterification presented the higher values (9.1% and 88.5% respectively). Compared to the MWAE and RFAE, the ultrasound assisted procedure allows to preserve the physico-chemical structure of the extracted pectin [19].

Therefore, in our study we conducted a series of PEC extractions from apple pomace, ultrasound assisted, by comparing two different organic acids solutions (acetic and citric), aiming at evaluating the impact of time and temperature on PEC quality (galacturonic acid content, methoxylation and esterification degree and electrostatic charge) and extraction yield. Additionally, the obtained pectin with higher galacturonic acid content and extraction yield were assessed biologically *in vitro* by using Neonatal Normal Human Dermal Fibroblasts (NHDF) for their potential to be used in biomedical applications. Our hypothesis was that the use of ultrasound could reduce the temperature for extracting pectin with high yield of the process, by preserving the intrinsic bioactive functionalities that can be exploited for several biomedical applications.

## 2 Materials and methods

### 2.1 Materials and chemicals

Glacial acetic acid (ACS reagent, ≥99.7%), citric acid (ACS reagent, ≥99.5%), hydrochloric acid (ACS reagent, 37%), ethanol 96%, sodium chloride (ACS reagent, ≥99.0%), phenol red (indicator ACS), sodium hydroxide (reagent grade, ≥98%), *m*-hydroxydiphenyl, D-(+)-Galacturonic acid monohydrate (analytical standard), sodium tetraborate, sulfuric acid (ACS reagent, 95.0–98.0%) and all other chemicals were purchased from Sigma-Aldrich, UK. Deionised water was obtained throughout Milli-Q® Water Purification System (IQ 7005, Merk, UK).

### 2.2 Apple biowaste processing and preparation

Apples (*Malus domestica* Bork) var. Royal Gala, from different origins (France, UK, South Africa, Chile), were purchased in a local supermarket. Subsequently, samples were visually verified to remove any damaged areas and hand-washed with tap water. Then, they were cut and ground using a fruit juicer (Cookworks, Argos, UK). The resulting pulp was passed through the juicer 3 more times to maximise the water removal and get smaller solid particles. Apple pomace yield in relation to whole apple and moisture content of apple pomace were determined by using the AOAC method [20], while the soluble solids from the extracted juice were measured by using a digital refractometer (RS PRO, UK).

Wet apple pomace was dried at 68 °C in a vacuum oven (SVAC1-2, SHEL LAB, UK) for 24 h before milling with an electric grinder (Blender LB20E, Waring Commercial, US) into powder and then, stored in grip seal bags in desiccator until further use.

### 2.3 Experimental design of the pectin extraction from the apple pomace

Extraction of pectin from apple pomace was carried out using a combination of variables including acidic solution from acetic acid (AA) or citric acid (CA), sonication time (25 or 50 min) by using an ultrasound water bath and temperature at 40 and 80 °C. The processing parameters were selected based on the most reported values in literature for successfully extracting pectin from other food waste biomasses [[21], [22], [23]].

Ultrasound assisted extraction was performed by mixing 15 g of apple pomace powder with 300 mL (to reach a ratio of 1 g /20 mL) of distilled water in which citric acid or acetic acid was added to reach a pH value of 1.5 by titration with 1 M HCl. The ultrasound water bath (USC 300T, VWR, UK) was set at 45 kHz, 80 W, and 100% amplitude. After sonication the mixture was centrifuged at 4400 rpm for 20 min (SORVALL ST 8R, Thermo-Fisher, UK), and the supernatant was collected, filtered using a nylon mesh, and transferred to standard glass flasks. Equal amount of ethanol was added to the supernatant and the resulting solution was kept for 24 h at 4–6 °C. Then, the precipitated pectin was centrifuged at 4400 rpm for 10 min and consecutively washed with ethanol while filtering through nylon mesh. The resulting pectin was dried at 45 °C on a heated incubator (MIR-162, Panasonic, Japan) until constant weight and kept and stored in grip seal bags in desiccator until further use.

The yield of the extracted pectin was calculated with the following formula (Eq. (1)):

(1) $$Pectin\;yield\;\left(\%\right)=\frac{dried\;pectin\;weight}{dried\;apple\;pomace\;weight}\times 100$$

### 2.4 Characterisation of the extracted pectin

#### 2.4.1 Determination of the anhydrouronic acid contents and the degree of methoxylation and esterification

The degree of methoxylation (DM) and anhydrouronic acid (AUA) contents and degree of esterification (DE) in pectin samples were analysed by conventional methods [24,25]. To 50 mg of pectin, 500 μL of ethanol, 10 mL of distilled water, 0.10 g NaCl and one drop of phenol red indicator were added. The solution was stirred for 15 min to dissolve all the components, and then titrated with 0.1 M NaOH until the colour changed (Titration A). Subsequently, 2.5 mL of 0.25 M NaOH was added to the mixture and allowed to stand for 30 min at room temperature. Finally, 2.5 mL of 0.25 M HCl was added, and the mixture was titrated again with 0.1 M NaOH until the colour turned red (Titration B). The degree of methoxylation was calculated by using the following equation (Eq. (2)):

(2) $$DM\left(\%\right)=\frac{meq\;Titration\;B\times 31\times 100}{weight\;of\;sample\;\left(mg\right)}$$

Where meq Titration B are the milliequivalents of NaOH used for the Titration B, and 31 is the molecular weight of the methoxyl group.

The anhydrouronic acid content was calculated according to the equation (3) (Eq. (3)):

(3) $$AUA\left(\%\right)=\frac{176\times 100}{z}$$

Where 176 is the molecular weight of AUA and z was calculated according to the equation (3) (Eq. (4)):

(4) $$z=\frac{weight\;of\;sample\;\left(mg\right)}{meq\;Titration\;A+meq\;Titration\;B}$$

Finally, the degree of esterification of the extracted pectin was calculated according to Equation (5) (Eq. (5)):

(5) $$DE\left(\%\right)=\frac{176\times DM\%\times 100}{31\times AUA\%}$$

#### 2.4.2 Galacturonic acid content analysis

A colorimetric method based on the *m*-hydroxydiphenyl reagent was used to measure the total galacturonic acid (GA) content of the extracted pectin following the protocol proposed by Gharibzahedi et al. [26]. Briefly, 500 μL of pectin solution (concentration of 200 μg/mL) was poured into a glass tube vial, and then 3 mL of sulfuric acid/sodium tetraborate was added and immediately cooled in a bath containing cold water. A continuous operation including shaking the tubes for 30 s with a vortex mixer (VORTEX 3, IKA, Germany), heating in a water bath (GLS Aqua 12 Plus, Grant, UK) at 100 °C for 5 min and cooling in ice water was performed. Then, 100 μL of *m*-hydroxydiphenyl (0.15% in 0.5% NaOH) were added to the vial and kept under shaking for 5 min (SSM1, Stuart, UK). Finally, the absorbance of the resulting solutions was read at 525 nm using a multiplate reader (FLUOstar Omega, BMG Labtech, Germany). For the preparation of the calibration curve, solutions of galacturonic acid (between 1 and 200 mg mL^−1^) were used.

#### 2.4.3 Molecular weight determination

The molecular weight of the extracted pectin was assessed by size-exclusion chromatography (SEC; 1260 Infinity GPC/SEC System, Agilent), equipped with a PL aquagel-OH MIXED-H 8 μm column. The samples were dissolved overnight at 2 mg/mL concentration in a recommended buffer (0.2 M NaNO_3_ + 0.01 M NaH_2_PO_4_ at pH 7), and, then, filtered through a 0.45 μm membrane (Titan 3, PTFE, ThermoScientific, UK) prior to injection (20 μl). The column set was calibrated with narrow pullulan standards and, thus, all molecular weight values were determined.

#### 2.4.4 ^1^H NMR measurement

The extracted pectin samples were analysed by NMR spectroscopy. Saturated samples were prepared in 0.7 mL D_2_O with TMSP-d4 [3-(trimethylsilyl)-2,2,3,3-tetradeuteropropionic acid] (Sigma-Aldrich, UK) added as an internal reference (0.0 ppm). The ^1^H NMR spectra were obtained at 80 °C on a Bruker Avance III HD 700 MHz NMR spectrometer using a Prodigy TCI cryoprobe. Each spectrum was acquired with 16 scans and 32 K datapoints (transformed to 128 K). Baseline corrections were applied before integration.

#### 2.4.5 Fourier transform infrared spectroscopy (FTIR-ATR)

FTIR-ATR spectroscopy analysis was performed on the extracted pectin. The infrared spectra were obtained with a spectrophotometer Spectrum one equipped with UATR accessory. The readings were taken in the wavelength range of 4000–550 cm^−1^, for each of the eight independent samples of each combination: acidic solution x sonication time x temperature, at least five consecutive readings were taken from pectin flakes. The average value was considered as representative for each sample.

#### 2.4.6 ζ -potential measurement

The ζ-potentials of pectin solutions (1:1 mg mL ^−1^) were measured by laser Doppler electrophoresis (Zetasizer Nano, Malvern instrument, US). Three sets of at least 10 measurements were averaged to get the final ζ-potential value for each PEC solutions.

#### 2.4.7 Rheological analysis

PEC solutions were solubilised in deionised water at 2% (w/w) under stirring at 25 °C for 16 h, then solutions were allowed to rest overnight at 4 °C prior to the rheological experiments. The tests were performed by using a stress-controlled rheometer (MCR302, AntonPaar GmbH, Graz, Austria) equipped with 25 mm parallel plate geometry. For each test, each pectin solution was poured on lower plate at 25 °C. De-hydratation was prevented by a water trap while temperature control was guaranteed with a Peltier system. The shear strain amplitude on each pectin solution was measured by the shear strain test at 25 °C (rotational oscillation 1 Hz, strain from 0.01% to 500%), while the frequency sweep test was performed using angular frequencies (ω) from 100 to 0.1 rad/s and a strain value within the linear viscoelastic region of 1%. Furthermore, the solution viscosity was determined using a shear rate from 0.1 to 100 1/s and a strain of 1%. Rheological tests were performed in triplicate.

#### 2.4.8 Pectin as biomaterials: *in vitro* cell tests

##### 2.4.8.1 Cell culture and seeding

Neonatal Normal Human Dermal Fibroblasts (NHDF) were purchased from Lonza Biosciences (Switzerland) and cultured as recommended by the seller. Briefly, fibroblasts were grown at 37 °C, 5% CO_2_, in Dulbecco's Modified Eagle Medium (DMEM, Sigma) supplemented with 10% fetal bovine serum (FBS), 2 mM l-glutamine and a 1% antibiotic mixture containing penicillin and streptomycin (100 U mL^−1^). To perform biocompatibility assays, PEC solutions at different concentrations (10, 25, 50, 100, 250, 500 and 1000 μg/mL) were prepared by dissolving the pectin powders in DMEM and then sterilised by filtration through a 0.22 mm Millex GP PES membrane syringe-driven filter unit (Millipore, SLS, UK) using 5 mL plastic syringes. Suspensions of 8 × 10^4^ cells and 10 × 10^4^ cells in DMEM were seeded on each well of a 96 and 48-multiwell plates respectively, with the different diluted PEC solutions, and then incubated with at 37 °C, 5% CO_2_ for the necessary biological tests.

##### 2.4.8.2 Cytocompatibility studies

Cell viability was assessed with the live/dead staining (LIVE/DEAD® Cell Imaging Kit, Life Technologies, Thermo Scientific, US) at 24 h in 48-multiwell plates. According to the manufacturer's protocol, membranes were washed with phosphate buffered saline (PBS, Sigma-Aldrich, UK) and stained with 150 μl solution of 4 μM Ethidium homodimer-1 and 2 μM calcein in PBS. After 30 min of incubation at room temperature, cells were imaged with a EVOS M5000 fluorescence microscope to detect calcein (ex/em 488 nm/515 nm) and Ethidium homodimer-1 (ex/em 570 nm/602 nm), respectively.

Furthermore, at the same time point, Presto Blue assay was exploited to test the metabolic activity of cells seeded with the different diluted PEC solutions in 96-multiwell plates. A Filter-based FLUOstar® Omega multi-mode reader (FLUOstar® Omega, Germany) was used to measure the fluorescence (560 nm excitation and 590 nm emission) after 1.30 h of incubation with a 10% aliquot of Presto Blue (Thermo Scientific, USA). Results were expressed as mean ± standard deviation.

Finally, the cell morphology was observed by nucleus and cytoskeleton staining after 48 h of cell seeding. Briefly, cells were fixed with 4% paraformaldehyde solution for 15 min, followed by three washing steps with PBS. Cells were then permeabilised using 0.1% v/v Tween20® in PBS for 5 min. Rhodamine-phalloidin was prepared using 1 : 100 dilution of phalloidin-tetramethylrhodamine B isothiocyanate (Sigma Aldrich, P1951) in 1% v/v Tween20® in PBS for 30 min, and then washed three times with PBS. One drop of DAPI (VECTASHIELD®) antifade mounting media was added to each sample, then covered with a glass slide and imaged using a EVOS M5000 fluorescence microscope.

#### 2.4.9 Statistical analysis

The analytical determination results were processed by one-way ANOVA, with mean separation by Tukey's test at 95% confidence level. A multifactor ANOVA was performed on the extraction parameters: Acid type (A), Extraction time (Et), Temperature (Tp) and their interactions, to evaluate their effects on the analytical determinations performed on the extracted pectin. The infrared information was analysed by Principal Component Analysis (PCA) to group the different extractions. PCA was used to reduce the dimensionality of the spectral data, which allowed to identify patterns and relationships in the data, useful for understanding the chemical properties of extracted pectin. The spectra were pre-processed to compensate and remove the bias linked to the experimental assessment by baseline correction (MicroLab Expert, FTIR Software, Agilent, US). Subsequently, different methods such as standard normal variance (SNV), multiplicative dispersion correction (MSC), and first and second derivatives were evaluated on the range of 1800–650 cm^−1^ of the spectra (known as fingerprint) that provided key information to differentiate samples from different treatments [27], which were run with the statistical software R Core Team, using the ChemoSpec package. Data processing was performed using Statgraphics Centurion 19 (Statpoint Technologies, Inc., USA) and R statistical software (version 3.6.3, R statistics, US).

## 3 Results

### 3.1 Analytical determination

In this work, Royal gala apples have been bought from a local store and they were characterised by soluble solids and moisture content, presenting 12.41 ± 0.62° Brix, and after removing the water from the pomace, dry matter represented 19.63 ± 0.43% apple pomace (dry base).

The effect of ultrasound-assisted extraction with the combination of different processing parameters (acid type, temperature, and time of extraction) on the analytical properties of the extracted pectin have been investigated in this work. These results are summarised in Table 1 and elaborated by the multifactor ANOVA to investigate if these variables were statistically significant or not (Table 2).

**Table 1:** Yield, galacturonic acid content, methoxylation and esterification degree, and ζ-potential of the extracted pectin samples from apple pomace obtained by conventional acidic extraction at pH = 1.5 with different temperatures and times. The values are shown as average ± SD.

Code	Acid	Temp. (°C)	Time (min)	Yield (%)	GalA (%)	DM (%)	DE (%)	ζ-potential (mV)
**CA40**-**25**	CA	40	25	3.1 ± 0.7	27.1 ± 4.8	41.4 ± 2.0	59.1 ± 1.3	−22.9 ± 1.1
**CA80**-**25**	CA	80	25	7.1 ± 1.9	31.0 ± 3.9	37.5 ± 2.1	63.0 ± 5.6	−13.2 ± 0.7
**AA40**-**25**	AA	40	25	1.2 ± 0.3	19.7 ± 0.5	33.8 ± 3.2	58.1 ± 1.9	−22.7 ± 2.8
**AA80**-**25**	AA	80	25	10.8 ± 2.9	43.9 ± 0.7	27.5 ± 0.8	58.0 ± 0.3	−16.1 ± 0.4
**CA40**-**50**	CA	40	50	5.0 ± 0.3	36.9 ± 2.0	36.6 ± 3.1	65.2 ± 4.4	−13.5 ± 1.2
**CA80**-**50**	CA	80	50	11.8 ± 1.5	43.7 ± 2.6	33.2 ± 1.4	61.4 ± 1.4	−15.8 ± 0.2
**AA40**-**50**	AA	40	50	8.6 ± 2.3	24.4 ± 1.4	32.0 ± 4.4	61.2 ± 5.2	−20.9 ± 0.8
**AA80**-**50**	AA	80	50	10.1 ± 2.4	49.2 ± 2.4	26.8 ± 1.7	58.2 ± 3.4	−18.6 ± 0.5
**SIG-APP**	–			–	67.0 ± 2.6	31.9 ± 1.3	58.9 ± 2.4	−24.1 ± 1.1

**Table 2:** F-ratio values and significance levels obtained in multifactor ANOVA for the physico-chemical parameters according to the factors: Acid type (A), Extraction time (E_t_), Temperature (T_p_) and their interactions.

	A	E_t_	T_p_	A x E_t_	A x T_p_	E_t_ x T_p_	A x E_t_ x T_p_
**Yield (%)**	1.37^NS^	17.18**	47.33***	0.0^NS^	0.01^NS^	2.72^NS^	11.49**
**GalA (%)**	0.72^NS^	239.36***	792.12***	35.67***	320.16***	2.95^NS^	1.44^NS^
**DM (%)**	21.5**	4.57*	10.11*	1.21^NS^	0.73^NS^	0.24^NS^	0.01^NS^
**DE (%)**	5.46*	1.72^NS^	0.19^NS^	0.06^NS^	0.24^NS^	3.18^NS^	0.74^NS^
**ζ-potential (mV)**	57.23***	12.49**	91.87***	18.94**	0.72^NS^	88.63***	19.79**

^NS^, not significant. *p < 0.05, **p < 0.01, ***p < 0.001.

The yield of the pectin obtained from the different extractions ranged from 1 to 12%, depending on the type of acid, time, and temperature of extraction. Particularly, it can be observed that the yield increased with increasing time and temperature. As example, for the citric acid, the yield increased from 3.1 ± 0.7% at 40 °C for 25 min to 11.8 ± 1.5% at 80 °C for 50 min of extraction. According to the F-ratio reported in Table 2, the temperature of extraction (*T*_*p*_) presented the highest influence (47.33 ***) followed by the extraction time (*E*_*t*_, 17.18 **) while the acid type (*A*) presented a statistical effect only in interaction with the other 2 processing factors (*A x E*_*t*_ *x T*_*p*_, 11.49 **). Thus, temperature was a crucial parameter, because its increase allowed the increase of pectin solubility, resulting in a higher yield. The behavior was described in literature in different works reporting extraction of pectin from different biomass [28,29].

The same substantial influence of the time and temperature of extraction was observed for the content of galacturonic acid (*E*_*t*_, 239.36*** and *T*_*p*_, 792.12***). GalA is the most prevailing building block of pectin, which makes its determination a very important step in the analysis of pectin's chemical structure [30]. The range of the analysed galacturonic acid was between ∼20 and 50% with the highest content found in the pectin extracted with acetic acid at 80 °C for 50 min. Commercial apple pectin purchased from Sigma-Aldrich was used as control and it was found to be characterised by 67% GalA within the range reported into the specification sheet of the supplier.

Furthermore, the degree of esterification (DE) is another parameter that affects pectin quality and applications. Indeed, according to the extraction conditions, different proportions of the acid groups of the GalA units are esterified and this is knows as DE [31]. Moreover, GalA units can be partly methoxylated, where the backbone presents methyl ester forms (–COOCH_3_), and this can be calculated as degree of methoxylation (DM) [32].

In our work, all the extraction conditions led to a DE ranging from 58 to 65% (intermediate AUA values are reported in Table S1). In contrast with the other analytical determinations, the use of the different acid type influenced the DE (5.46 *) where the citric acid extractions provided the highest values (at 80 °C for 25 min, 63.0 ± 5.6% for CA respect with 58.0 ± 0.3% for AA). As shown in Table 1, both acids presented a similar DE to that of commercial SIG-APP (58.9 ± 2.4%). Numerous researchers described that the pectin solubilisation into the solvent happened due to the breakage of the plant cell wall under the influence of the ultrasound [33,34]. Particularly, ultrasound is a green method that rises the selectivity, decreases reaction time, and encourages macro- and micro-mixing via acoustic cavitation, creating cavities/bubbles. After collapse, these can release huge amounts of energy that is made available to break the structure where pectin is contained [35]. As demonstrated by Zhang et al. [36] high intensity ultrasound (up to 300 W cm^−2^) can increase the DE >70%. They reported a similar value of DE close to 60% when using a lower ultrasound power (∼60 W cm^−2^) at 20 °C for 30 min.

Then, according to the DM, pectin can be categorised as high methoxy pectin (DM > 50%) and low methoxy (DM < 50%) [37]. Table 1 shows that the DM values of all the apple pectin were in the ranges of ∼27–41%; thus, our pectin can be classified as low methoxyl. Moreover, the pectin with the highest DM was obtained with the citric acid by comparing the same extraction conditions (time and temperature) of the acetic acid. This trend was confirmed by the F-ratio (21.5**). These considerations on the DM are important for selecting the use of the pectin in biomedical application as bioink and hydrogel for tissue engineering and regenerative medicine. Particularly, low or high DM require different conditions for crosslinking pectin. Pectin with low DM is characterised by high number of free carboxyl groups with high cation-binding ability. The binding of divalent cations e.g. Ca^2+^, Mg^2+^ produces junction zones between two polyguluronate chain dimers. These segments present an “egg-box” structure, where the binding of the cation to the carboxyl groups of two opposite pectin chains was stabilised by van der Waals interactions and hydrogen bonds [38]. Thus, our extracted pectin in all the conditions can be suitable to manufacture bioprinted constructs or *in situ* gelling systems. Indeed, the ζ-potential values ranging from −13 to −24 mV confirmed the presence of a high number of free COOH groups, fundamental for the further ionotropic gelation with divalent ions. Furthermore, this negative charge of the extracted pectin can allow to use it as polyelectrolyte (specifically as polyanion) for the surface functionalisation of medical devices by technique of Layer-by-Layer (LbL) assembly. LbL is an environmental-friendly technique that allows to create a multilayered coating at the nanoscale, exploiting the electrostatic interaction of polyelectrolytes, for modifying the surface topography and/or entrapping biomolecules/drugs to impart specific biological activities [39,40].

**Fig. 1:**
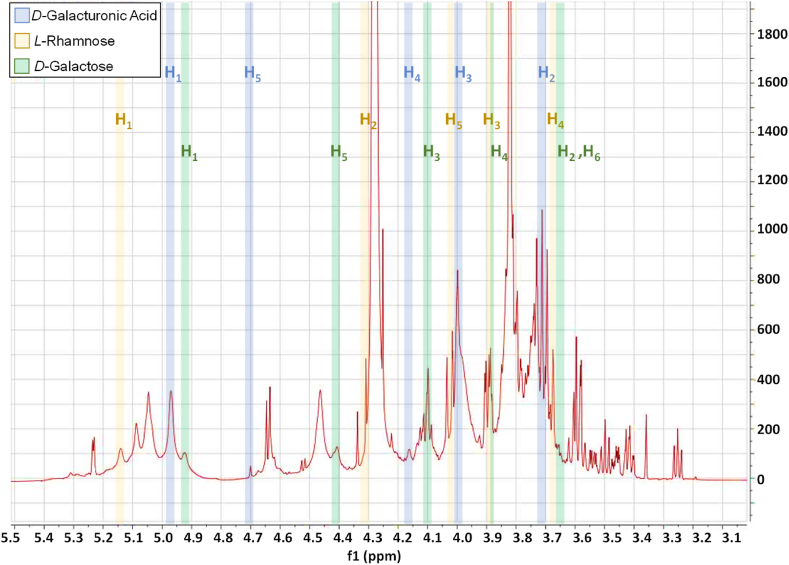
^1^H NMR spectrum of pectin from apple pomace extracted by using acetic acid at 80 °C for 25 min ultrasound-assisted.

^1^H NMR spectra of the extracted and commercial apple pectin were compared. All the spectra were characterised by a broad signal chain (i.e. CH_3_ and CH_2_ groups) ranging from 0 to 2.5 ppm [41] (Figure S1). Particularly, signals at 2.11 and 1.91 ppm are from the –COCH_3_ groups located at 3-*O*- and 2-*O*-galacturonic acid. Then, signals at 1.30 ppm and 1.27 ppm are from the CH_3_ group of *l**-*rhamonose. The peak at 3.92 ppm is derived from the CH_3_ group that is associated with the carboxyl groups of GalA. The remaining pectin signals are assigned to the 5 protons found in GalA (H_1_, 4.97 ppm; H_2_, 3.73 ppm; H_3_, 3.97 ppm; H_4_, 4.16 ppm, and H_5_, 4.70 ppm) (labelled in blue in Fig. 1 and reported in Table 3). Furthermore, signals at 5.13 ppm and 4.92 ppm located in the anomeric region are assigned to H_1_ Rha and H_1_ Gal, respectively. Furthermore, the extracted pectin showed differences compared with the control SIG-APP. Indeed, the acetyl groups of GalA acid and methyl groups of Rha were not visible in the ^1^H NMR spectrum at range 2.5–1 ppm (Figure S1). Also, the extracted pectin showed a visible increase in the intensities of the peaks at 4.92 ppm of the H_1_ Gal, that could overlap the peek at 4.97 ppm of H_1_ GalA, and at 4.70 ppm of the H_5_ GalA. However, all the other protons, characterising the GalA [42], were less intense or not detected.

**Table 3:** ^1^H NMR chemical shifts of pectin from apple pomace extracted by using acetic acid at 80 °C for 25 min ultrasound-assisted.

	H_1_	H_2_	H_3_	H_4_	H_5_	H_6_
***d******-*galacturonic acid**	4.97	3.73	3.97	4.16	4.70	n.d.
***l******-*rhamonose**	5.13	4.31	3.88	3.67	4.02	n.d.
***d******-*galactose**	4.92	3.64	4.10	3.87	4.41	3.66

n.d. = not detected.

**Fig. 2:**
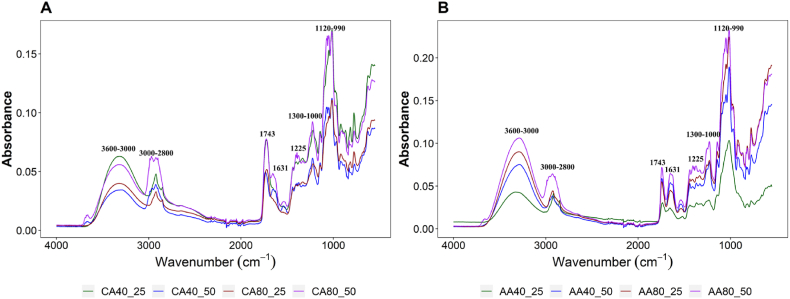
ATR-FTIR spectra with baseline correction of the apple pectin samples obtained in the mid-infrared 4000-650 cm^−1^ range after acidic extraction with citric (**A**) and acetic acid (**B**).

FTIR-ATR pectin spectra obtained after acidic extraction for all the different treatments are illustrated in Fig. 2A and B. The main absorption peaks recorded around 3600-3000 cm^−1^ were caused by O–H stretching, while characteristic absorption peak of pectin-reproduced polysaccharides due to C–H stretching of CH_2_ groups was observed between 3000 and 2800 cm^−1^ [18,43]. Stretching vibration (C=O) of methyl-esterified and carboxylate ions (free carboxyl groups) of pectin resulted in the bands at 1743 cm^−1^ and 1631 cm^−1^, respectively [44]. The tendency of increasing intensities and band area of esterified carboxyl groups may indicate an increase in degree of esterification [45]. Certainly, esterified carboxyl groups exhibit an increasing trend in their intensities and band areas, as esterification degree value increases [46]. Also, the higher absorbance for esterified carboxylic groups, compared to free carboxylic groups, would indicate a higher degree of esterification [47].

Bands related to the stretching of the C–O bond were observed between 1300 and 1000 cm^−1^ [43], while the absorption band at 1225 cm^−1^ was due to the cyclic C–C bond in the ring structure of pectin. Finally, the region between 1120 and 990 cm^−1^ has been reported for the spectral identification of galacturonic acid in peptide polysaccharides [48]. Also, the bands around 1049–50 and 953-58 cm^−1^ in pectin spectra may be attributed to the presence of arabinogalactan and rhamnogalacturonan molecules in the hairy regions of the polysaccharides [49].

Furthermore, some more considerations can be done on mid-infrared (MIR) spectra where the wavenumber range of 4000–650 cm^−1^ can be classified into two different regions: functional group (4000-1500 cm^−1^) and fingerprint region (1500-650 cm^−1^). In both regions, changes in absorbance values are observed due to the different treatments (Fig. 2A and B). However, differences in the fingerprint region are more evident in peaks of interest such as those associated with the degree of esterification (743 cm^−1^ and 1631 cm^−1^), gallic acid (1120-990 cm^−1^) and pectin structure cycle (1300-1000 cm^−1^). Therefore, the chemometric analysis of the spectrum was performed within information in the region of 1800–650 cm^−1^wavenumbers.

**Fig. 3:**
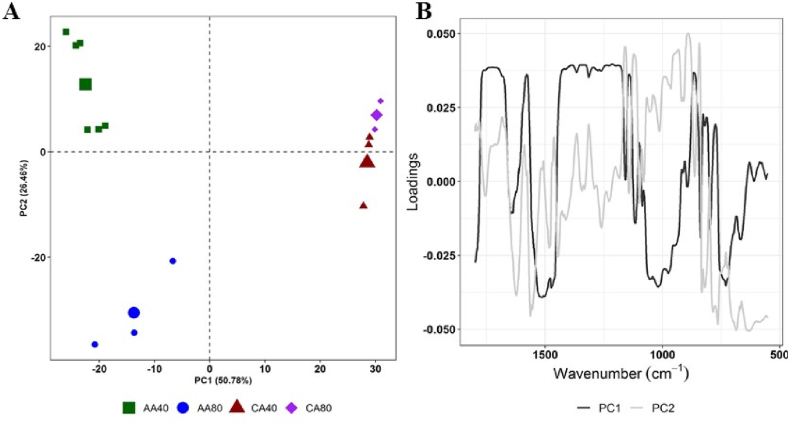
(A) PCA of the processed infrared signal spectra of extracted pectin (25 min) with baseline correction + MSC normalisation; (B) Pectin apple spectrum and loadings for PC1 and PC2.

Following the baseline correction, an exploratory PCA analysis was performed with the information of the region between 1800 and 650 cm^−1^ for 25-min treatments, using different processing techniques such as SNV, MSC, and first and second derivatives. The best clustering results were evidenced with MSC, which are illustrated in Fig. 3A and B. The first four principal components (PC) explained 92.41% of MIR variability, where the first and second principal components provided the main contribution of the samples variability (50.78% and 26.46% for PC1 and PC2 respectively) (Fig. 3A), while the third and fourth components (PC3, PC4) explained lower than 10% each of the PC analysis (8.17 and 6.98% respectively, as shown in Figure S2A and B).

The scatter plot shows three different groups according to the treatment applied: (i) AA40, (ii) AA80 and (iii) CA40 and CA80. This shows a clear effect on pectin composition when temperature is varied from 40 to 80 °C in the acetic acid extraction, while this effect is not observed with citric acid. Thus, FTIR analyses confirmed the influence of temperature during the extraction on the pectin structure and the content of GAs, which were lower in AA40 than those obtained in AA80 (Table 1), which could explain the differences evidenced by the analysis of the IR spectra.

The loadings plot for the first two components indicates that the region between 1800 and 1700 cm^−1^ and 1420 to 1180 cm^−1^ are strongly associated with the samples in grouped PC1 positive region, where CA40 and CA80 samples were located. As discussed above, these regions are associated with the degree of esterification and C–O stretching, respectively. This is consistent with the significant higher degree of esterification for the samples extracted with citric acid (Table 1, Table 2) An important contribution from the region between 1200 and 900 cm^−1^, it is also evident in the negative part of PC1 where most of the AA40 samples were observed. This zone could be influenced by the presence of galacturonic acid in the pectin, and as shown in Table 1, where these samples presented the lowest concentrations of galacturonic acid. Finally, AA80 samples were grouped in PC2 negative region, loadings plot evidence a considerable contribution of the band 1631and 1565 cm^−1^. The peak at 1636-1606 cm^−1^ indicated (C=O) stretching vibration of carboxylate ion. The ratio of the area of the peak at 1743 cm^−1^ (COO-R) to the sum of the areas of the peaks at 1743 cm^−1^ and 1636 cm^−1^ (COO^−^) can be used to quantify the degree of esterification [18].

**Fig. 4:**
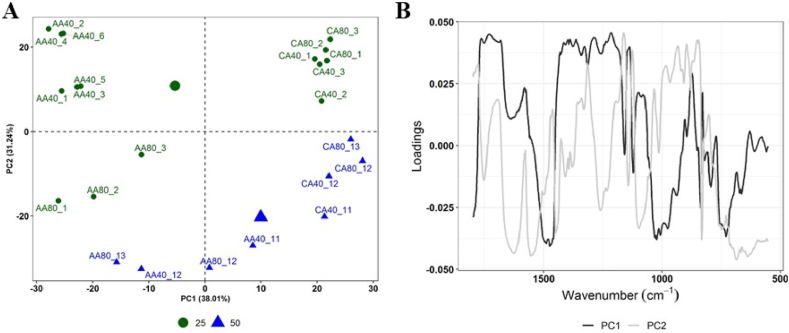
(A) PCA of all processed infrared signal spectra of extracted pectin (25 and 50 min) with baseline correction + MSC normalisation; (B) Pectin apple spectrum and loadings for PC1 and PC2.

Furthermore, when comparing data from pectin spectra extracted at 25 and 50 min, PCA analysis evidenced the effect of the extraction time on the chemical characteristics of the pectin, which allows their aggrupation (Fig. 4A). Samples obtained at 50 min (quadrant IV, samples CA80) and pectin obtained at 25 min (quadrant I, samples CA40 and CA80) were grouped in the positive region of PC1. The wavenumber between 1800 and 1650 cm^−1^ (associated with the esterification degree) and 1400-1100 cm^−1^ shows an important contribution for the separation of this type of samples (Fig. 4B), which is consistent with the higher degrees of esterification of the samples extracted with CA at 25 and 50 min (Table 1). Samples obtained at 25 min on the negative region of PC1 (quadrant II pectin AA40; quadrant III pectin AA80), are mostly associated with the 1100 -900 cm^−1^wave numbers, influenced by galacturonic acid. Finally, pectin obtained at 50 min distributed over the negative region of PC2 were associated with bands at 1631 cm^−1^, 1550 cm^−1^, 1450 cm^−1^, 1250 cm^−1^, and 1100 cm^−1^. These latter regions can be associated with C–O–C stretching of the glycosidic bond at ∼1144 cm^−1^, and C–C or C–O stretching (at 1050 and 1019 cm^−1^ respectively) in the monosaccharide ring, that represents the main characteristic bands of pectic-type polysaccharides [49]. The bands at 1600-1620 cm^−1^ may be related to the asymmetric stretching vibration of the carboxylate anion (COO-). Then, the band at 1440-1460 cm^−1^ is associated with the (O=C–O) vibration of the non-esterified groups of pectin and the presence of a peak at 1420 cm^−1^, typical of the (COO-) of pectic acid salts, is visible [50].

Finally, the molecular weight is a key-parameter for evaluating the relationship between polysaccharide structure and function [51], where its value is associated with the pectin gelling properties, fundamental for being considered suitable for the manufacturing of hydrogels in tissue engineering [52] The M_W_ of the extracted pectin samples ranged from 1.314 to 1.511 × 10^5^ Da, the number average molecular weight (M_n_) ranged from 1.095 to 1.114 × 10^5^ Da with a consequent poly-dispersity index ranged between 1.2 and 1.4. Representative molecular weight distribution for AA40-50 pectin sample is reported in Figure S3, where the peak was eluted at retention time ∼18 min. The commercial pectin had similar value (1.13 × 10^5^ Da) in accordance with the literature [53]. Therefore, no differences have been noticed among all the extracted pectin samples, with variation of type of organic acid, and temperature and time of US-assisted extraction.

### 3.2 Rheological analysis

**Fig. 5:**
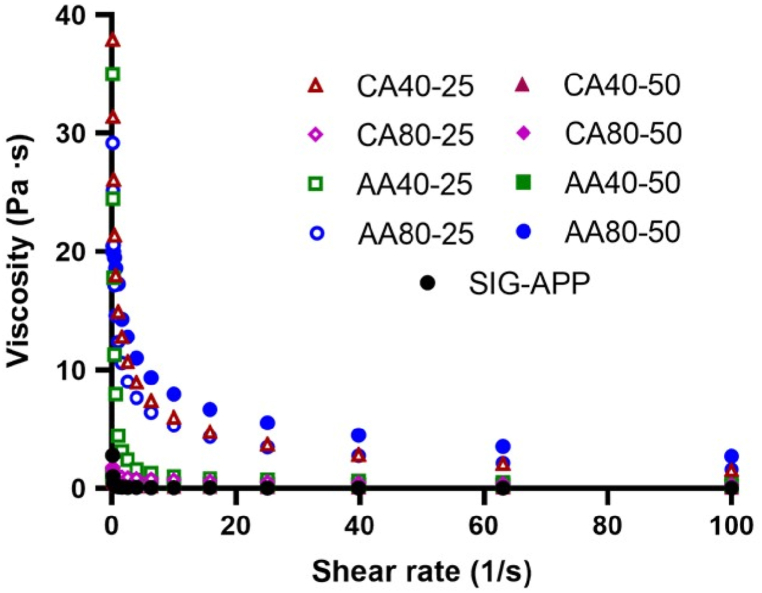
Flow-curves of the extracted pectin solutions from different acidic conditions. Apple pectin from Sigma-Aldrich (SIG-APP) has been used as control.

**Fig. 6:**
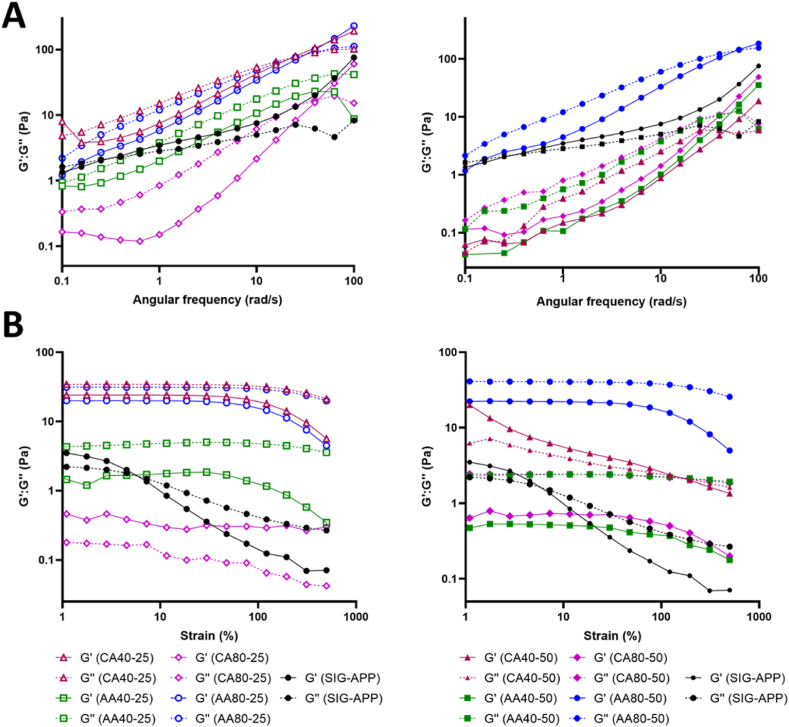
Rheological properties of pectin solutions obtained from (**A**) frequency and (**B**) strain sweep tests after 25 (left) and 50 (right) mins of extraction. Apple pectin from Sigma-Aldrich (SIG-APP) has been used as control.

Rheological analysis measurements reported a different behaviour for extracted pectin solutions from the apple pomace compared to commercial pectin from Sigma-Aldrich (SIG-APP). Flow curves revealed a lower viscosity for SIG-APP compared to extracted pectin solutions (Fig. 5) while the frequency sweep tests at 25 °C showed an opposite trend of G′ and G’’ (Fig. 6A) having a SOL state (G’ < G″) for extracted pectin solutions and a GEL state for SIG-APP one (G’ > G″). Strain sweep tests allowed to identify the linear viscoelastic region (LVE) which indicates the range in which the test can be carried out without destroying the structure of the sample. LVE is visible in all the extracted pectin (except for CA 40–50) reaching a yield point for strain up to 50% (Fig. 6B). On the other hand, the SIG-APP solution shows a narrow LVE with a yield point at 5% strain. Furthermore, rheological measurements highlighted the effect of extraction process on the mechanical behaviour of pectin solutions. Indeed, the use of AA or CA strongly influenced the properties of the final solutions, higher viscosity was obtained when the extraction process was performed using AA at 80 °C (AA80-25 and AA80-50) while for CA a reduction of the viscosity was observed increasing the temperature and the time (Fig. 5). All the tested conditions maintained a SOL state at 25 °C however differences in the frequency and strain sweep test plots were observed ascribed to the acidic conditions (CA or AA) used within the extraction process (Fig. 6). When CA was used, G′ and G″ decreased for the higher temperature while the longer time reduced the stability of the solutions to strain exhibiting a lower yield point. On the contrary, for AA the process at 80 °C guaranteed higher G′ and G″ values compared to 40 °C, however the extraction time did not affect the mechanical properties of the solutions tested, indeed only a slight reduction of G′ and G″ values was observed for AA40-50 compared to AA40-25.

The tested pectin solutions show G′ and G″ values of few Pa, highlighting the potential of this material to be applied in the field of soft tissue engineering and regenerative medicine as the mechanical properties of several human tissues are in the range from few Pa to kPa [54].

### 3.3 *In vitro* cell tests

Neonatal Normal Human Dermal Fibroblasts were seeded on the tissue culture plates with different concentrations of the extracted and commercial pectin to assess their cytocompatibility for biomedical applications, particularly for tissue engineering and regenerative medicine.

**Fig. 7:**
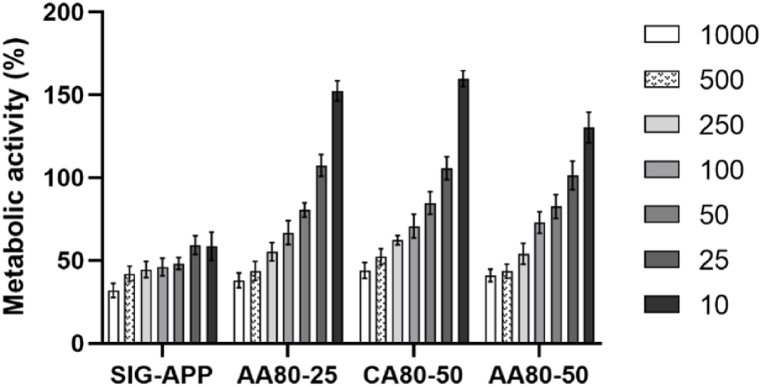
Metabolic activity of Neo-dermal fibroblast cells after 48 h of seeding in presence of different concentration (from 1000 to 10 μg/mL) of the extracted pectin. Apple pectin from Sigma-Aldrich (SIG-APP) has been used as control. The results are shown as average ± SD after normalisation to the control of cells seeded on TCPs.

**Fig. 8:**
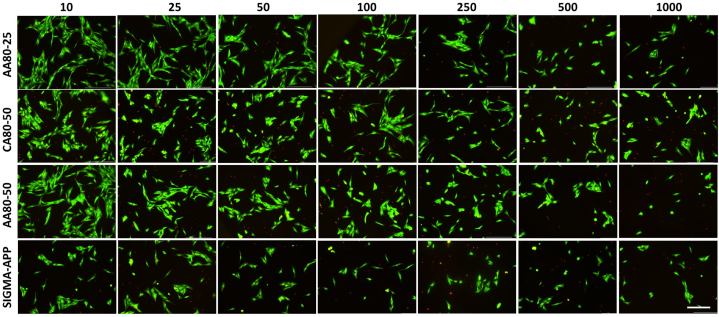
Live/dead images of Neo-dermal fibroblast cells after 24 h of seeding in presence of different concentration (from 1000 to 10 μg/mL) of the extracted pectin. Commercial apple pectin purchased from Sigma-Aldrich (SIG-APP) has been used as control. Scale bar = 300 μm.

The NHDF metabolic activity was assessed by using Presto Blue assay (Fig. 7) after 48 h, showing a significant increase when the concentration of the dissolved pectin is below 250 μg/mL, confirming the results observed by the live/dead staining assay (Fig. 8). Interestingly, the AA80-25 and CA80-50 exhibited the highest metabolic activity of the NHDF when compared to the remaining sample AA80-50 (at 1000 μg/mL p < 0.01). However, all the samples containing the extracted pectin encouraged the growth and a quicker spreading of the cells. In contrast, a significant viability reduction was observed on the cells seeded with the commercial pectin. After 48 h, a reduction of more than 50% compared to the other samples was detected at concentrations in the range of 10–50 μg/mL. Furthermore, the viability of the NHDF was assessed by live/dead staining assay after 48 h of seeding, as shown in Fig. 8. Lower concentrations showed a high cell viability and ability to promote cell attachment. NHDF showed the typical elongated and flattened morphology on all the extracted pectin samples and spreading homogeneously along the TCP surface. On the other hand, highest concentrations seemed to have affected the cell behavior. Particularly, from the concentration of 500 μg/mL, it was noticed different dead cells (labelled in red) mainly for the samples AA80-50 and SIG-APP.

**Fig. 9:**
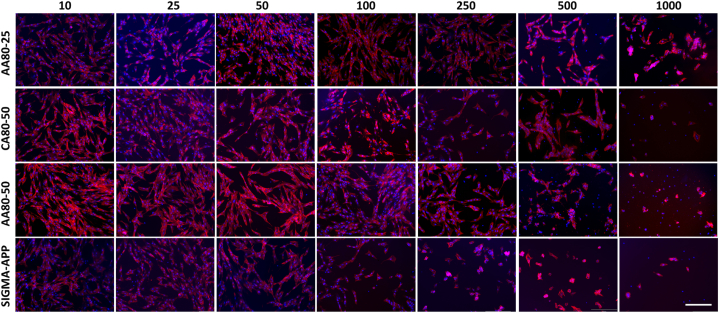
Immuno-staining images of Neo-dermal fibroblast cells after 48 h of seeding in presence of different concentration (from 1000 to 10 μg/mL) of the extracted pectin. Commercial apple pectin purchased from Sigma-Aldrich (SIG-APP) has been used as control. Scale bar = 300 μm.

Immunostaining assays confirmed the previous results with the cell maintained spindle-shape in the presence of low concentrations of the extracted pectin, while cells at higher concentrations evidenced a rounded shape and cellular contraction with smaller nucleus (Fig. 9). This can be related with the cytotoxic effect of pectin confirmed by low metabolic activity detected by Presto Blue assay and Live/Dead staining.

## 4 Conclusion

A comprehensive comparison between different processing factors of a combined organic acidic and ultrasound-assisted extraction applied to obtain pectin from apple biowaste was made to evaluate the procedure performance, including yield and physico-chemical properties, to propose an alternative methodology to the mineral acidic extraction. We found in this work that temperature and time mainly influenced the properties of the extracted pectin in terms of extraction yield, GalA content and methoxylation degree, where temperature presented the highest influence on the process. Moreover, we observed that the acid type only showed effect on the ζ-potential of the extracted materials. Considering the highest cytocompatibility of the extracted pectin compared with the commercial one, the evaluated procedure with the assisted ultrasound condition allows to obtain materials, capable to preserve the bioactive functionality and can be proposed for different biomedical applications, including as hydrogels for soft tissue engineering and regenerative medicine, thanks to the low moduli measured through rheology, and as polyelectrolyte for the development of multilayered coating to modify the surface of medical devices and/or to allow the controlled release of biological molecules and drugs.

## Author contribution statement

Joel Girón-Hernández, Piergiorgio Gentile: Conceived and designed the experiments; Performed the experiments; Analysed and interpreted the data; Contributed reagents, materials, analysis tools or data; Wrote the paper. Michelle Pazmino, Corinne Wills: Performed the experiments; Analysed and interpreted the data; Wrote the paper. Yeison Fernando Barrios-Rodríguez, Chiara Tonda Turo: Performed the experiments; Analysed and interpreted the data; Contributed reagents, materials, analysis tools or data; Wrote the paper. Fabio Cucinotta, Maria Benlloch-Tinoco: Analysed and interpreted the data; Contributed reagents, materials, analysis tools or data. Data availability statement: Data will be made available on request. Declaration of interest's statement: The authors declare that they have no known competing financial interests or personal relationships that could have appeared to influence the work reported in this paper.

## Declaration of competing interest

The authors declare that they have no known competing financial interests or personal relationships that could have appeared to influence the work reported in this paper.

## Appendix A Supplementary data

Supplementary material.docx Supplementary material.docx
