# Supplementary material for: Exploring the effect of utilising organic acid solutions in ultrasound-assisted extraction of pectin from apple pomace, and its potential for biomedical purposes
Source: Heliyon. 2023 Jun 28;9(7):e17736. doi: 10.1016/j.heliyon.2023.e17736 (PMC10336596; doi:10.1016/j.heliyon.2023.e17736)
Supplement: Supplementary material.docx [file mmc1.docx]

**Supplementary material**

**Exploring the effect of utilising organic acid solutions in ultrasound-assisted extraction of pectin from apple pomace, and its potential for biomedical purposes**

Joel Girón-Hernández, Michelle Pazmino, Yeison Fernando Barrios-Rodríguez, Chiara Tonda Turo, Corinne Wills, Fabio Cucinotta, Maria Benlloch-Tinoco, Piergiorgio Gentile

**Table S1.** Anhydrouronic acid content (AUA(%)) of the extracted pectin samples from apple pomace obtained by conventional acidic extraction at pH= 1.5 with different temperatures and times (values shown as average ± SD)

| **Code** | **Acid** | | **Temp. (°)** | | **Time (min)** | **AUA** **(%)** |
| --- | --- | --- | --- | --- | --- | --- |
| **CA40-25** | CA | | 40 | | 25 | 66.75±0.08 |
| **CA80-25** | CA | | 80 | | 25 | 49.56±0.11 |
| **AA40-25** | AA | | 40 | | 25 | 24.02±0.05 |
| **AA80-25** | AA | | 80 | | 25 | 27.26±0.12 |
| **CA40-50** | CA | | 40 | | 50 | 69.24±0.09 |
| **CA80-50** | CA | | 80 | | 50 | 69.03±0.08 |
| **AA40-50** | AA | | 40 | | 50 | 37.67±0.08 |
| **AA80-50** | AA | | 80 | | 50 | 30.57±0.07 |
| **SIG-APP** | - |  | |  | | 54.63±0.01 |

**
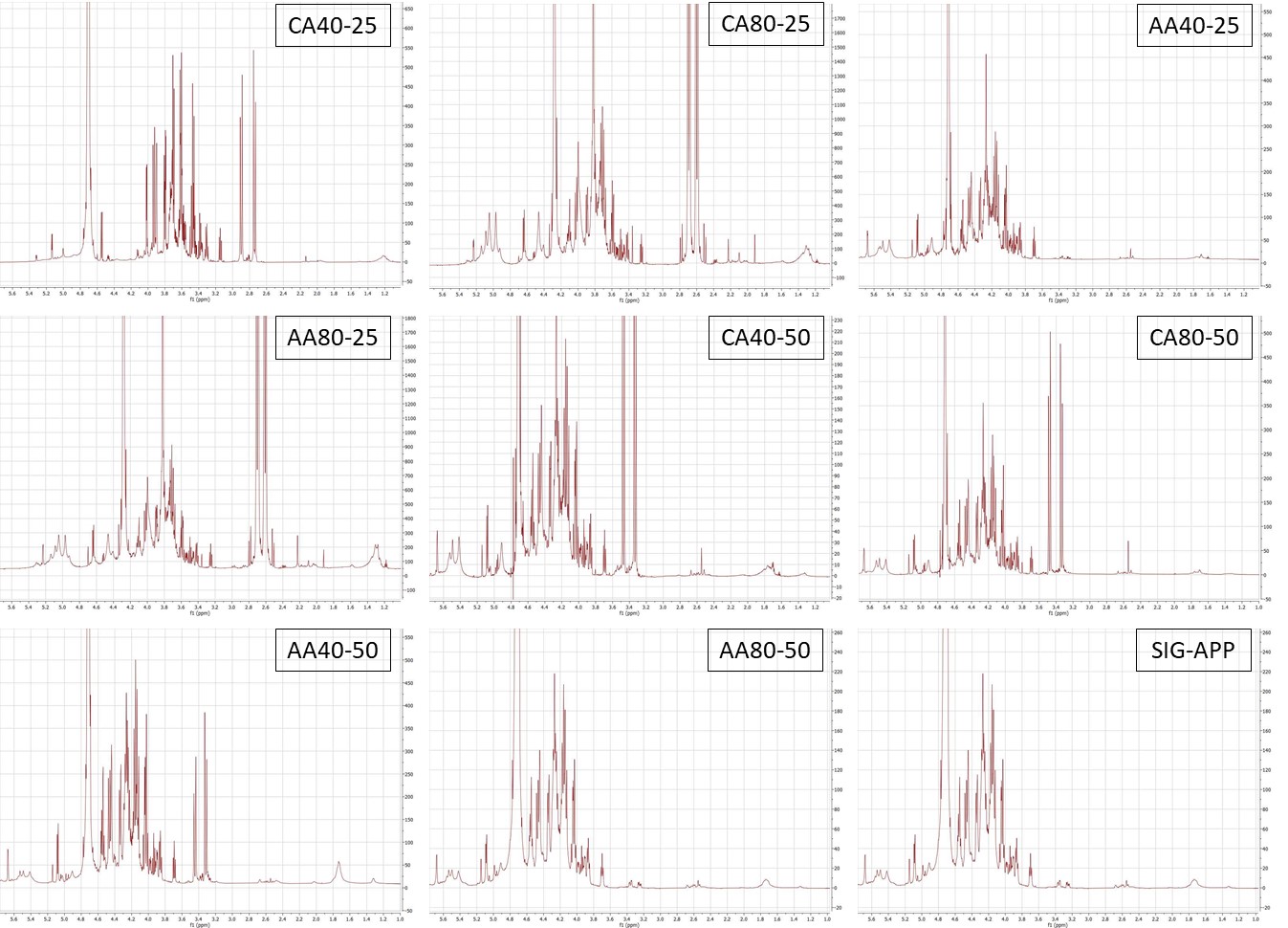
**

**Figure S1**. ^1^H NMR spectra of pectin from apple pomace extracted by using the different processing conditions ultrasound-assisted.


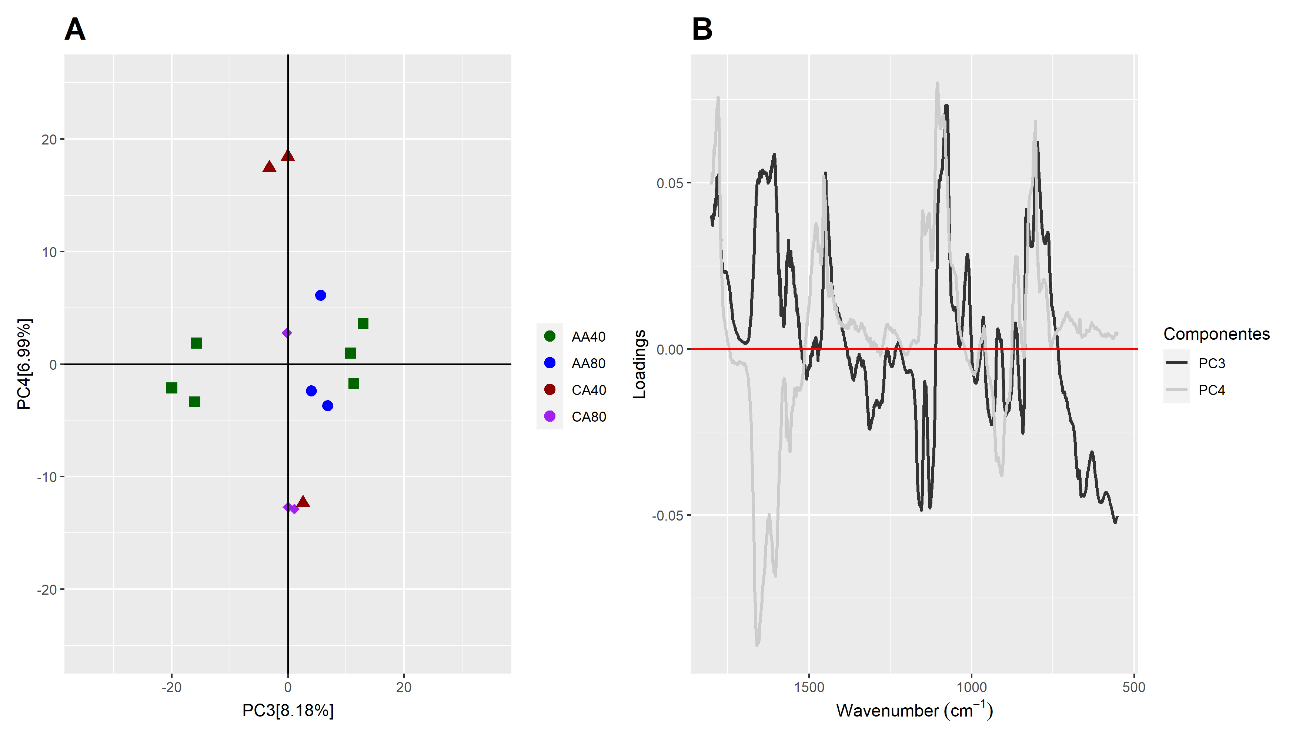


**Figure S2.** (**A**) PCA of the processed infrared signal spectra of extracted pectin (25 min) with baseline correction + MSC normalisation; (**B**) Pectin apple spectrum and loadings for PC3 and PC4.

**Figure S3**. SEC data reporting molecular weight distribution for AA40-50 pectin sample
